# Ultrasound-Assisted sequential processing of barley straw using binary acidic and hydrated ternary deep eutectic solvents for nanocellulose production^☆^

**DOI:** 10.1016/j.ultsonch.2025.107376

**Published:** 2025-05-05

**Authors:** Dileswar Pradhan, Swarna Jaiswal, Brijesh K. Tiwari, Amit K. Jaiswal

**Affiliations:** a School of Food Science and Environmental Health, Faculty of Sciences and Health, Technological University Dublin - City Campus, Central Quad, Grangegorman, Dublin, Ireland; b Centre for Sustainable Packaging and Bioproducts, Technological University Dublin - City Campus, Central Quad, Grangegorman, Dublin, Ireland; c Sustainability and Health Research Hub, Technological University Dublin - City Campus, Grangegorman, Dublin, Ireland; d Health Engineering & Materials Science Research Hub, Technological University Dublin - City Campus, Grangegorman, Dublin, Ireland; e Teagasc Food Research Centre, Ashtown, Dublin, Ireland

**Keywords:** Nanocellulose, Lignin, Agriculture waste, Deep eutectic solvent, Ultrasound

## Abstract

This study aimed to valorise barley straw by using a binary acidic deep eutectic solvent (DES) made from choline chloride and lactic acid for biomass pretreatment, and a hydrated ternary DES (HDES) composed of betaine, oxalic acid, and water (BOW HDES) for downstream processing to produce nanocellulose. The ultrasound-assisted DES pretreatment significantly enhanced lignin and hemicellulose solubilisation, achieving an average lignin removal of 70.54 % and hemicellulose solubilisation of 69.58 % under optimal conditions. Purification of US-DES-treated solid residue resulted in a cellulose yield of 39.81 ± 1.47 % with a purity of 91.31 ± 0.93 %, comparable to or exceeding conventional fractionation methods. The yield of lignin-rich material was 9.40 ± 0.89 % with a lignin purity of 83.29 ± 1.57 %. Further, nanocellulose was produced using a sequential process comprising low-viscosity HDES treatment, which improved fibre swelling and solubilisation, followed by high-intensity ultrasound (HIUS) treatment for nanoscale defibrillation. DLS analysis of the optimal nanocellulose sample revealed that 77.8 % of nanoparticles had a diameter below 100 nm, demonstrating a high yield of nanoscale material. XRD analysis confirmed the preservation of the cellulose I crystalline structure throughout processing, ensuring structural integrity. These findings demonstrate an efficient and sustainable biorefinery approach for lignin, cellulose, and nanocellulose extraction from agricultural residues, offering potential for scalable nanocellulose production.

## Graphical abstract

**Figure:**
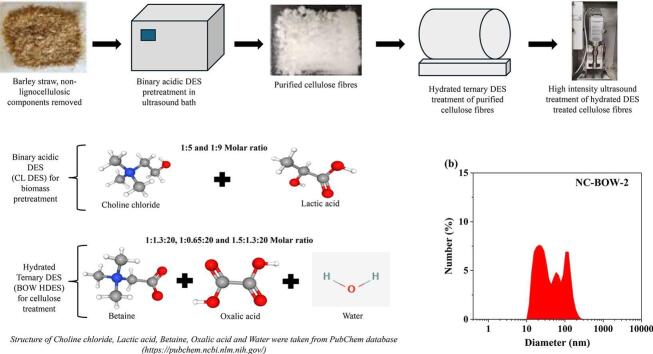

## 1 Introduction

Nanocellulose is a versatile and promising natural material with at least one dimension in the nanometre range. It is primarily classified into two categories: cellulose nanofibers (CNF) and cellulose nanocrystals (CNC) [1]. According to ISO/TS 20477:2023 “Nanotechnologies — Vocabulary for cellulose nanomaterial”, nanocellulose is defined as materials where at least one dimension is between 1 nm and 100 nm [2]. In recent years, its potential applications have been extensively investigated across various sectors, including food packaging [3], pharmaceuticals and biomedical materials [4], electronics [5], and water purification [6]. Given its wide applicability, there is a growing need to explore novel lignocellulosic biomass sources and develop economically viable and sustainable nanocellulose production methods to meet industrial demand and drive innovation.

In recent years, the scientific community has shown significant interest in cereal residues as renewable biomass resources for nanocellulose production [7,8]. Among these, barley straw is produced in large quantities annually due to extensive barley cultivation. Global barley production reached approximately 146 million tonnes in 2021, according to the United Nations Food and Agriculture Organization (FAO). The abundance of this agricultural residue presents an opportunity for valorisation into high-value nanocellulose products.

Barley straw is particularly promising due to its high lignocellulosic content. A study by Duque et al. [9] reported that barley straw comprises 39.1 % cellulose, 25.7 % hemicellulose, and 16.4 % lignin, making it a suitable feedstock for nanocellulose extraction in a biorefinery setting. Given its availability, composition, and potential for sustainable processing, barley straw represents a cost-effective and eco-friendly alternative to conventional cellulose sources.

In the biorefining process, biomass pretreatment is a critical step that disrupts the lignin-carbohydrate complex, facilitating the extraction of individual components for conversion into high-value products [10]. Among various pretreatment approaches, deep eutectic solvents (DES) have emerged as promising green solvents for the fractionation of lignocellulosic biomass due to their biodegradability, low toxicity, and tunability [11,12].

DES are synthesised by mixing two components (binary DES) or three components (ternary DES) in appropriate molar ratios. Their composition always includes at least one hydrogen-bond acceptor (HBA) and one hydrogen-bond donor (HBD), which interact through hydrogen bonding to form a stable eutectic mixture [13]. Choline chloride (ChCl), a commonly used HBA, can be combined with various acidic HBDs, such as oxalic acid, lactic acid, citric acid, formic acid, or malic acid, to synthesise acidic DES [14]. Among these, ChCl-lactic acid (CL DES) has demonstrated high efficiency in biomass pretreatment across multiple studies [[15], [16], [17]].

Furthermore, the effectiveness of CL DES can be significantly enhanced by integrating ultrasound technology, which improves mass transfer, accelerates lignin and hemicellulose solubilisation, and enhances process efficiency. The combination of ultrasound-assisted DES pretreatment has shown potential in reducing energy consumption while improving biomass fractionation, making it a promising approach for sustainable nanocellulose production.

Mechanical defibrillation techniques, such as ultrasonication, high-pressure homogenisation, and ball milling, are commonly employed to reduce cellulose fibres to the nanoscale. However, these methods alone often result in high energy consumption and inconsistent product quality. To address these challenges, chemical or biological pretreatment of cellulose can enhance nanocellulose extraction, improving both yield and properties [18].

In recent years, the use of green, biodegradable solvents, such as deep eutectic solvents (DES), has been investigated for nanocellulose production [[19], [20], [21], [22]]. While DES-based methods offer environmental and economic advantages, their high viscosity and cost remain key challenges. To mitigate these issues, incorporating water as a third component in the DES system has been shown to reduce viscosity, lower processing costs, and enhance cellulose-solvent interactions [23]. The application of hydrated deep eutectic solvents (HDES) for nanocellulose production has been explored in recent studies, demonstrating their potential to improve processing efficiency and reduce energy requirements [23,24].

To the best of our knowledge, the ultrasound-assisted pretreatment of barley straw using choline chloride-lactic acid (CL DES) has not yet been explored. Additionally, the application of hydrated deep eutectic solvent (HDES), formulated from betaine, oxalic acid, and water (BOW HDES), has not been investigated for nanocellulose production. Given these research gaps, this study aims to develop an ultrasound-assisted DES (US-DES) pretreatment method for barley straw using CL DES and evaluate its effectiveness in biomass fractionation. Following pretreatment, the recovery of lignin and recycling of DES were conducted to assess the sustainability of the process. The cellulose-rich solid material (CRSM) obtained after US-DES pretreatment was further purified to yield high-purity cellulose fibres (PCF). Subsequently, BOW DES was synthesised at three different molar ratios and applied to PCF treatment, followed by high-intensity ultrasound (HIUS) processing to facilitate nanocellulose production. This study provides insights into a novel biorefinery approach, integrating ultrasound-assisted DES pretreatment and HDES processing for the efficient and sustainable production of nanocellulose from barley straw.

## 2 Experimental Section

### 2.1 Materials

The barley straw biomass used in this study was purchased from Dublin, Ireland. All chemicals were of analytical grade and obtained from Merck Ireland. The reagents used included lactic acid (CAS: 50–21-5), choline chloride (CAS: 67–48-1), calcium carbonate (CAS: 471–34-1), ethanol (CAS: 64–17-5), sulphuric acid (CAS: 7664–93-9), oxalic acid (CAS: 144–62-7), and sodium hypochlorite (CAS: 7681–52-9). For composition analysis, high-performance liquid chromatography (HPLC)-grade sugar standards were used, including D-(+)-glucose (CAS: 50–99-7), D-(+)-xylose (CAS: 58–86-6), D-(+)-mannose (CAS: 3458–28-4), D-(+)-galactose (CAS: 59–23-4), and L-(+)-arabinose (CAS: 5328–37-0).

### 2.2 Biomass composition analysis

The structural carbohydrates (cellulose and hemicellulose) and lignin content in the biomass were analysed following the National Renewable Energy Laboratory (NREL, USA) protocol. Biomass hydrolysis was conducted in a water bath at 30 °C for one hour using 72 % (w/w) sulphuric acid. After hydrolysis, the sample was diluted to 4 % acid concentration and autoclaved at 121 °C for one hour. Once cooled, the hydrolysate was vacuum-filtered using a filtration crucible, and the hydrolysis liquor was collected for further analysis.

The acid-insoluble residue (AIR) retained in the filtration crucible was dried in an oven for 24 h before being ashed in a muffle furnace at 575 °C for 24 h to determine the acid-insoluble lignin (AIL) content. To quantify acid-soluble lignin (ASL), a 10 mL solution was prepared by diluting 0.5 mL of hydrolysis liquor with 9.5 mL of deionised water (dilution factor = 20). The absorbance of this solution was measured at 280 nm using a UV–visible spectrophotometer. The ASL content was calculated using a dilution factor of 20, an absorptivity value of 84.8 L g^−1^ cm^−1^, and a path length of 1 cm. The total lignin content in the biomass was reported as the sum of AIL and ASL.

To determine monosaccharide composition, 10 mL of hydrolysis liquor was taken, and its pH was adjusted to 6 using calcium carbonate. The solution was then filtered through a 0.2 μm syringe filter and transferred into an HPLC sample vial. Analysis was performed using ultra-high-performance liquid chromatography (UHPLC), equipped with a refractive index detector (RID) and a Biorad Aminex HPX-87P column. The mobile phase was ultrapure water, filtered through a 0.2 μm membrane and degassed before use. Each sample was analysed with a 20-minute run time, injecting 10 µL per run at a flow rate of 0.6 mL/min. The column and RID temperatures were maintained at 80 °C and 35 °C, respectively. Glucose concentration was reported as cellulose content, while hemicellulose content was calculated as the sum of xylose, arabinose, galactose, and mannose present in the sample.

### 2.3 Biomass sample preparation

The raw barley straw (RBS) was ground and sieved to obtain a particle size below 500 μm. The removal of ethanol- and water-soluble non-lignocellulosic components was performed following the method described in our previous study [25]. An appropriate amount of RBS was mixed with ethanol (1:10 w/v ratio) and treated in an ultrasonic bath at 40 °C and 45 kHz for 30 min. After treatment, the solid and liquid fractions were separated by centrifugation at 4000 × g for 10 min. The ethanol was recovered using a rotary evaporator, while the solid fraction was resuspended in deionised water (1:10 w/v ratio) and subjected to a second ultrasonic treatment under the same conditions. Following treatment, the solid and liquid fractions were again separated by centrifugation, and the solid fraction was washed with deionised water before being freeze-dried for further processing. This processed material was referred to as extractives-free barley straw (EFBS) [25].

### 2.4 Pretreatment of biomass

#### 2.4.1 DES preparation

Deep eutectic solvents (DES) were synthesised by combining choline chloride (ChCl) and lactic acid (LA) at two distinct molar ratios: 1:5 (CL-A) and 1:9 (CL-B). The required amounts of each component were weighed and added to a Duran glass bottle according to the specified molar ratios. The mixture was then heated on a hot plate at 60 °C with continuous stirring at 300 rpm until a clear liquid was formed. After synthesis, the DES was allowed to cool and subsequently stored in sealed glass bottles for future use. Both CL-A and CL-B DES remained stable at room temperature, maintaining their liquid form.

#### 2.4.2 US-DES pretreatment

US-DES pretreatment of extractives-free barley straw (EFBS) was performed following the method described in our previous study [25]. The process was conducted in an ultrasound bath at frequencies of 25 kHz and 45 kHz. The ultrasonic bath was designed to operate at these two frequencies only. The EFBS sample was mixed with the CL-A or CL-B at a solid-to-DES ratio of 1:20 w/w, and the treatment was carried out at 80 °C for 3 or 5 h. The maximum controllable temperature of 80 °C in the ultrasonic bath was used across all experiments. Furthermore, the experiments were limited to a maximum duration of 5 h to reduce processing time and overall energy consumption of the process [25].

Following pretreatment, the solid residue and liquid fraction were separated by centrifugation at 4000 × g for 10 min. The solid residue, referred to as cellulose-rich solid material (CRSM), was washed three times with pure ethanol, followed by three washes with deionised water [26]. The wash liquids were combined with the liquid fraction from the DES pretreatment, which was later utilised for lignin recovery and DES recycling. The CRSM was freeze-dried for composition analysis, while the never-dried CRSM was retained for further purification.

The efficiency of pretreatment was evaluated based on hemicellulose solubilisation (Equation (1) and lignin solubilisation (Equation (2):

(1) $$Hemicellulose\;solubilisation\;\left(\%\right)=\;\left(\frac{\left(H_{I}\times W_{I}\right)-\left(H_{R}\times W_{R}\right)}{H_{I}\times W_{I}}\right)\times 100$$

(2) $$Lignin\;\;solubilisation\;\left(\%\right)=\;\left(\frac{\left(L_{I}\times W_{I}\right)-\left(L_{R}\times W_{R}\right)}{L_{I}\times W_{I}}\right)\times 100$$

Where $W_{I}$ is the initial weight (g) of the EFBS sample, $W_{R}$ is the weight (g) of solid residue obtained after US-DES treatment, $H_{I}$ is the hemicellulose content (%) in EFBS sample, $H_{R}$ is the hemicellulose content (%) in solid residue, $L_{I}$ is the lignin content (%) in the EFBS sample, and $L_{R}$ is the lignin content (%) in the solid residue.

#### 2.4.3 DES and lignin recovery

The recovery of DES and lignin from the liquid fraction (containing DES, ethanol, and water) obtained during US-DES pretreatment was adapted from the methods reported by [27,28], with modifications based on our previous study [25]. Initially, ethanol was recovered from the liquid fraction using a rotary evaporator (Rotavapor® R-100, Buchi, Switzerland) at 45 °C under a low pressure of 150 mbar. After ethanol removal, the remaining liquid phase (DES and water) was diluted with deionised water to achieve a total water volume five times the amount of the initial DES used for pretreatment. The mixture was then left at room temperature for 48 h, allowing lignin precipitation. The lignin-rich material (LRM) was recovered by centrifugation at 4000 × g for 10 min, washed twice with distilled water, and freeze-dried [25]. The LRM yield, relative to the initial EFBS biomass weight, was calculated using Equation (3). The purity of lignin, defined as the percentage of lignin in the total solids of the LRM, was determined following the lignin estimation method described in Section 2.2. The lignin recovery was calculated using Equation (4), as reported by Hou et al. [29]. Following lignin recovery, the waste liquid was collected for DES recycling. Water was removed using a rotary evaporator at 55 °C under a low pressure of 65 mbar, and the DES recovery efficiency was determined using Equation (5).

(3) $$LRM\;yield\;\left(\%\right)=\;\left(\frac{Weight\;of\;recovered\;LRM\;(g)}{Initial\;weight\;of\;EFBS\;biomass\;(g)}\right)\times 100$$

(4) $$Lignin\;recovery\;\left(\%\right)=\;\left(\frac{Lignin\;amount\;in\;LRM\;(g)}{Lignin\;amount\;in\;EFBS\;biomass\;(g)}\right)\times 100$$

(5) $$DES\;recovery\;\left(\%\right)=\;\left(\frac{Mass\;of\;DES\;recovered\;(g)\;}{Initial\;mass\;of\;DES\;used\;for\;pretreatment\;(g)}\right)\times 100$$

#### 2.4.4 Cellulose purification

The cellulose-rich solid material (CRSM), obtained under the optimal conditions of US-DES pretreatment, was purified using a 2 % sodium hypochlorite solution following the method described in our previous study [25]. The purification was conducted at a solid-to-liquid ratio of 1:10 w/v, a treatment temperature of 60 °C, and a treatment duration of 60 min in a water bath. After the treatment, the white cellulose fibres were recovered by centrifugation at 4000 × g for 10 min. The fibres were then washed three times with deionised water and freeze-dried to determine their yield and purity [25]. The purity of the purified cellulose fibres (PCF) was evaluated following the biomass composition analysis protocol described in Section 2.2.

### 2.5 Nanocellulose production

#### 2.5.1 HDES preparation

Ternary hydrated deep eutectic solvents (HDES) were prepared by mixing betaine, oxalic acid, and water at different molar ratios (provided in Table 1). The required quantities of each component were weighed and added to a Duran glass bottle, followed by heating on a hot plate at 50 °C with continuous stirring at 300 rpm until a clear solvent was formed. The synthesis process was completed within 10 min, after which the HDES was stored in sealed glass bottles for further use.

**Table 1:** Molar ratios of three components of BOW HDES.

HDES code	Molar ratios of three components of HDES (B:O:W)		
	Betaine (B) (MW: 117.148 g/mol)	Oxalic acid (O) (MW: 90.03 g/mol)	Water (W) (MW: 18.01 g/mol)
BOW-1	1	1.3	20
BOW-2	1	0.65	20
BOW-3	1.5	1.3	20

#### 2.5.2 HDES treatment of cellulose

The HDES treatment of PCF was conducted in an autoclave, with treatment conditions optimised based on preliminary experiments. The experimental parameters varied included treatment temperature (100 and 120 °C), ramp-up time (20 and 25 min), holding time (30, 60, 90, and 120 min), and cooling time (20 and 25 min). The cellulose-to-HDES ratio was maintained at 1:25 w/v for all experiments, based on prior optimisation. Following treatment, the solid and liquid fractions were separated by centrifugation at 4000 × g for 10 min. The cellulose residue was then washed twice with water. Notably, further washing caused the supernatant to become turbid, indicating the presence of nanoparticles. To minimise nanoparticle loss, washing was limited to two cycles. The cellulose residue was subsequently freeze-dried to determine the recovery efficiency after HDES treatment, which was calculated using Equation (6). The wet cellulose residue was used for further nano-fibrillation via the high-intensity ultrasound (HIUS) process.

(6) $$C_{R}=\frac{C_{\mathrm{PHT}}}{C_{I}}\times 100$$

Where $C_{R}$ is the recovery of PCF (%) following the HDES treatment, $C_{\mathrm{PHT}}$ is weight of PCF recovered after HDES treatment, and $C_{I}$ weight of initial amount of PCF utilised for HDES treatment.

#### 2.5.3 HIUS treatment of HDES treated cellulose

The HDES-treated cellulose was suspended in deionised water for nano-fibrillation using the high-intensity ultrasound (HIUS) process. The amount of water added was adjusted to maintain a cellulose concentration of 0.1 wt%, calculated based on the recovery efficiency of cellulose after HDES treatment. Only the cellulose samples treated under optimal HDES conditions were selected for nano-fibrillation. The cellulose-water mixture was processed using a HIUS system (Hielscher, Germany) equipped with a 2 cm probe, operating at 1000 hdT and 20 kHz with an output power of 500 W. The treatment was conducted for 20 min. A portion of the nanocellulose sample was freeze-dried for X-ray diffraction (XRD) analysis. The utilisation of HIUS treatment for nanocellulose production has been reported in several studies [7,[30], [31], [32]].

### 2.6 Characterization

#### 2.6.1 Dynamic light scattering (DLS)

The particle size distribution, polydispersity index (PDI), and zeta potential of the nanocellulose samples were determined using dynamic light scattering (DLS). The analysis was performed with a Zetasizer Nano ZS instrument (Malvern Panalytical, USA), which operates at a scattering angle of 173° and a temperature of 25 °C. The PDI provides insight into the uniformity of the particle size distribution, while zeta potential indicates the surface charge and colloidal stability of the nanocellulose suspension. Prior to analysis, the nanocellulose suspension was diluted tenfold to prevent multiple scattering effects and ensure accurate measurements.

#### 2.6.2 Scanning electron microscopy (SEM)

The morphological characteristics of the biomass samples, at various stages of nanocellulose production, were examined using a desktop scanning electron microscope (SEM, Phenom™ XL). This instrument operates at an accelerating voltage of 10 kV, which provides sufficient resolution to observe surface topography and structural changes in the samples. The powdered samples were carefully mounted on carbon tabs to ensure good conductivity, and images were acquired at a magnification of 500 × .

#### 2.6.3 Fourier transform infrared spectroscopy (FTIR)

Fourier transform infrared (FTIR) spectroscopy was conducted to identify functional groups and analyse chemical structure modifications in the samples during the different stages of processing. The analysis was performed using an ATR-FTIR spectrometer (Nicolet iS5, Thermo Scientific, USA), which allows direct sample measurement without additional preparation. Spectra were collected in transmittance mode, recording 64 scans at a resolution of 4 cm^−1^ over a wavenumber range of 4000–400 cm^−1^. FTIR spectra were used to identify characteristic peaks corresponding to cellulose, hemicellulose, and lignin functional groups, providing insights into structural transformations resulting from DES and HDES treatments.

#### 2.6.4 X-ray diffraction (XRD)

The XRD analysis of the samples was performed using a Rigaku Miniflex benchtop X-ray diffractometer, equipped with a monochromatic CuKα radiation source (λ = 1.54059 Å, 40 kV, 15 mA). A 2θ angle range of 5° to 40°, a step width of 0.02°, and a scan speed of 2°/min were utilized as the scanning parameters. Segal's method (Equation (7) was utilized to calculate the crystallinity index (CI) of the samples [33,34].

(7) $$CI\;\left(\%\right)=\frac{I_{200}-{\;I}_{am}}{I_{200}}\times 100$$

I_200_ is the maximum intensity (in arbitrary units) at a 2θ angle around 22° and I_am_ is the minimum diffraction intensity in the same units at a 2θ angle around 18° [34,35].

### 2.7 Statistical analysis

All statistical analyses were performed using SPSS software (Version 29, IBM, USA). Experimental measurements were conducted in triplicate unless otherwise stated, and results are reported as mean ± standard deviation. Data were analysed using one-way analysis of variance (ANOVA) to assess significant differences between sample groups, followed by Tukey’s post hoc test for pairwise comparisons. A p-value < 0.05 was considered statistically significant.

### 2.8 Life cycle assessment

The life cycle assessment (LCA) study was conducted using the SimaPro (Release 9.6.0.1) software. The goal of the LCA study was to evaluate the environmental impacts of optimised nanocellulose production process. A cradle-to-gate LCA approach was employed where the scope of the study included the environmental impacts associated with resource extraction of materials such as electricity, water, and chemicals used in process. The environmental impact associated with the generation of barley straw was excluded from the scope of this LCA study. The LCA was conducted in a laboratory scale; therefore, the functional unit was kept as production of 2 g of PCF from barley straw. The system boundary of the LCA study and the inventory data is shown in Fig. S1. The impact associated with the chemicals, energy and other resources utilised in the process were obtain from the Ecoinvent 3 database provided in SimaPro software. Further, the life cycle impact assessment was carried out using the Environmental Footprint 3.1 (adapted) method.

## 3 Results and Discussion

### 3.1 Pretreatment of barley straw

In our previous study [25], the lignocellulosic composition of barley straw was determined, with the raw biomass containing 42.50 ± 0.25 % cellulose, 26.60 ± 0.32 % hemicellulose, 18.26 ± 1.01 % lignin, and 2.60 ± 0.05 % ash. Additionally, 10.04 ± 1.64 % of non-lignocellulosic components were present. Following the removal of these non-lignocellulosic components, the resulting extractives-free barley straw (EFBS) had an improved composition of 47.30 ± 0.52 % cellulose, 26.19 ± 0.04 % hemicellulose, 22.66 ± 0.28 % lignin, and 2.69 ± 0.80 % ash. In addition, the non-lignocellulosic components in the EFBS sample were 1.17 ± 1.02 %. The significant reduction in non-lignocellulosic components in the EFBS sample affected the proportions of other biomass components, such as cellulose, hemicellulose, lignin, and ash [25].

#### 3.1.1 US-DES pretreatment

The composition analysis of the solid fraction recovered after US-DES treatment using CL-A DES and CL-B DES is presented in Table 2, Table 3, respectively. The average solid recovery for the CL-A DES-treated samples ranged from 67 % to 78 %, whereas for the CL-B DES-treated samples, it ranged from 62 % to 69 %. The lower solid recovery in the CL-B DES-treated samples suggests that a higher acid concentration in DES enhances biomass solubilisation, leading to greater removal of non-cellulosic components.

**Table 2:** Composition of solid residue obtained after US-DES treatment at 80 °C using CL-A DES (ChCl:LA of 1:5 M ratio).

Sample code	Ultrasound Frequency (kHz)	Time (hours)	Solid Recovery (%)	Cellulose (%)	Hemicellulose (%)	Lignin (%)	Ash (%)	Lignin solubilisation (%)	Hemicellulose solubilisation (%)
CL-A-1	25	3	78.03 ± 0.98^b^	56.52 ± 0.57^a^	18.12 ± 0.20^c^	20.89 ± 0.37^b^	3.59 ± 0.14^a^	28.06 ± 0.38^a^	46.01 ± 1.27^a^
CL-A-2	25	5	74.85 ± 0.95^b^	60.36 ± 0.75^b^	16.32 ± 0.13^b^	19.16 ± 0.70^b^	3.61 ± 0.12^a^	36.68 ± 3.12^a^	53.35 ± 0.22^b^
CL-A-3	45	3	75.98 ± 0.98^b^	57.90 ± 0.80^ab^	16.97 ± 0.17^b^	21.23 ± 0.67^b^	3.72 ± 0.18^a^	28.80 ± 3.15^a^	50.77 ± 0.15^b^
CL-A-4	45	5	67.16 ± 1.04^a^	65.91 ± 0.60^c^	14.43 ± 0.18^a^	15.94 ± 0.56^a^	3.64 ± 0.23^a^	52.78 ± 0.93^b^	62.99 ± 1.03^c^

*Different letters in the same column indicate statistically significant differences (P < 0.05); n = 2.

**Table 3:** Composition of solid residue obtained after US-DES treatment at 80 °C using CL-B DES (ChCl:LA of 1:9 M ratio).

Sample code	Ultrasound Frequency (kHz)	Time (hours)	Solid Recovery (%)	Cellulose (%)	Hemicellulose (%)	Lignin (%)	Ash (%)	Lignin solubilisation (%)	Hemicellulose solubilisation (%)
CL-B-1	25	3	69.24 ± 0.70^b^	62.44 ± 0.68^a^	14.81 ± 0.80^a^	17.10 ± 0.24^c^	5.05 ± 0.25^a^	47.74 ± 1.27^a^	60.82 ± 2.52^a^
CL-B-2	25	5	64.84 ± 0.78^a^	66.03 ± 0.84^b^	13.75 ± 0.77^a^	13.43 ± 0.18^b^	6.16 ± 0.41^ab^	61.59 ± 0.06^c^	65.96 ± 2.31^a^
CL-B-3	45	3	64.27 ± 0.51^a^	64.53 ± 0.28^ab^	14.14 ± 0.99^a^	15.61 ± 0.71^c^	5.34 ± 0.47^ab^	55.74 ± 1.68^b^	65.28 ± 2.70^a^
CL-B-4	45	5	62.59 ± 0.70^a^	69.13 ± 0.80^c^	12.73 ± 0.62^a^	10.66 ± 0.58^a^	6.95 ± 0.55^b^	70.54 ± 1.92^d^	69.58 ± 1.13^a^

*Different letters in the same column indicate statistically significant differences (P < 0.05); n = 2.

Regardless of ultrasonic frequency, solid recovery decreased with increasing treatment time. However, a significant reduction was observed when treatment time increased from 3 to 5 h at 45 kHz for CL-A DES-treated samples and at 25 kHz for CL-B DES-treated samples. The minimum solid recovery (62.59 ± 0.70 %) was obtained for biomass treated with CL-B DES at 45 kHz for 5 h (CL-B-4 sample), indicating that higher acid concentration combined with prolonged ultrasound exposure maximised solubilisation efficiency.

Several studies have reported similar trends in solid recovery following DES treatment with ChCl-LA mixtures at different molar ratios. Chourasia et al. [36] conducted DES treatment (1:5 ChCl:LA, 12 h, 80 °C) on sugarcane bagasse, achieving solid recovery between 60.33 % and 65.67 %. Similarly, Oh et al. [37] reported an average solid recovery of 54.6 % when treating pine wood powder with DES (1:2 ChCl:LA, 130 °C, 6 h). In contrast, Yu et al. [38] found that *Triarrhena lutarioriparia* treated with DES at 90 °C for 3 h yielded 89.35 % (1:2 ChCl:LA) and 78.79 % (1:10 ChCl:LA). These variations indicate that solid recovery is influenced by biomass type, DES composition, temperature, and treatment duration.

A significant increase in cellulose content was observed following US-DES pretreatment compared to the EFBS sample (47.3 %). The average cellulose content in the CL-A DES-treated samples ranged from 56 % to 65 %, while in the CL-B DES-treated samples, it increased further to 62 % to 69 %. This higher cellulose enrichment in the CL-B DES-treated samples indicates that a greater proportion of acid in DES enhances the removal of non-cellulosic components, leading to more effective fractionation of biomass.

Irrespective of the ultrasonic frequency used, the cellulose content increased with longer treatment times for both CL-A and CL-B DES-treated samples. However, when the treatment time was held constant at 3 h, increasing the ultrasonic frequency from 25 to 45 kHz did not result in a significant change in cellulose content. In contrast, at a constant treatment time of 5 h, increasing the frequency from 25 to 45 kHz led to a notable increase in cellulose content in both CL-A and CL-B DES-treated samples. The highest cellulose content (69.13 %) was observed in the CL-B-4 sample (45 kHz, 5 h of treatment time), indicating that higher acidity, extended treatment duration, and increased ultrasonic frequency contribute synergistically to effective cellulose purification.

Similar enhancements in cellulose content following DES pretreatment have been reported in previous studies. Oh et al. [37] observed a significant increase in glucan content from 39.7 % (untreated) to 60.4 % (DES-treated) after applying DES (1:2 ChCl:LA, 130 °C, 6 h) to pine wood powder. Likewise, Yu et al. [38] reported that *Triarrhena lutarioriparia* treated with DES (1:2 ChCl:LA, 90 °C, 3 h) exhibited a cellulose content increase from 43.39 % to 48.3 %, while treatment with a 1:10 ChCl:LA DES raised it further to 54.66 %. These findings reinforce that DES composition, treatment duration, and process conditions significantly influence cellulose enrichment in lignocellulosic biomass.

Following US-DES treatment, a significant reduction in hemicellulose content was observed compared to the EFBS sample (26.19 %). The hemicellulose content in CL-A DES-treated samples ranged from 14.43 % to 18.12 %, whereas in CL-B DES-treated samples, it was further reduced to 12.73 % to 14.81 %. The lower hemicellulose content in CL-B DES-treated samples suggests that higher acid concentration in DES enhances hemicellulose solubilisation, making CL-B DES more effective at hemicellulose removal.

No major significant differences in hemicellulose content were observed with variations in treatment time and ultrasonic frequency. However, a gradual decrease in hemicellulose content was noted with an increase in treatment time for both DES types. The minimum hemicellulose content (12.73 %) was recorded in the CL-B-4 sample, which was treated at 45 kHz for 5 h, indicating that longer treatment durations and higher ultrasonic frequencies promote enhanced hemicellulose removal.

These findings are consistent with previous research. Oh et al. [37] reported a substantial reduction in hemicellulose content, from 22.1 % (untreated) to 10.6 % (DES-treated), after applying DES (1:2 ChCl:LA, 130 °C, 6 h) to pine wood powder. The comparable decrease in hemicellulose content observed in this study reinforces the effectiveness of US-DES treatment in selectively solubilising hemicellulose, thereby enriching the cellulose fraction.

The hemicellulose solubilisation efficiency for CL-A DES-treated samples ranged from 46.01 % to 62.99 %. A significant increase in hemicellulose solubilisation was observed when treatment time was extended from 3 to 5 h while keeping the ultrasonic frequency constant at either 25 kHz or 45 kHz. Similarly, when treatment time was held constant (3 or 5 h) and the ultrasonic frequency was increased from 25 to 45 kHz, a notable improvement in hemicellulose solubilisation was observed in CL-A DES-treated samples. These findings suggest that both treatment duration and ultrasonic frequency play a crucial role in disrupting hemicellulose structures, facilitating enhanced solubilisation.

In the case of CL-B DES-treated samples, the average hemicellulose solubilisation efficiency was higher, ranging from 60.82 % to 69.58 %. However, unlike CL-A DES-treated samples, no significant difference in solubilisation efficiency was observed with changes in treatment time or frequency. The maximum hemicellulose solubilisation efficiency (69.58 %) was recorded in the CL-B-4 sample, confirming that higher acid concentration in DES improves hemicellulose dissolution, but ultrasonic frequency and time variations have a lesser impact in highly acidic conditions.

The hemicellulose solubilisation efficiency obtained in this study was comparable to or higher than those reported in previous research. Yu et al. [38] conducted DES treatment at 90 °C for 3 h and reported hemicellulose removal of 17.43 % (1:2 ChCl:LA) and 40.44 % (1:10 ChCl:LA) from *Triarrhena lutarioriparia*. Similarly, Zheng et al. [39] achieved close to 50 % hemicellulose removal from passion fruit husks by applying DES (1:2 ChCl:LA) at 120 °C for 3 h. These findings highlight that US-DES treatment can significantly improve hemicellulose solubilisation, particularly when optimised DES compositions and process conditions are used.

The lignin content in the solid fraction recovered after US-DES treatment varied between 15.94–20.89 % for CL-A DES-treated samples and 10.66–17.10 % for CL-B DES-treated samples. No significant differences in lignin content were observed among the CL-A-1, CL-A-2, and CL-A-3 samples, suggesting that under these conditions, the lignin removal efficiency reached a plateau. However, in the CL-B DES-treated samples, increasing the treatment time from 3 to 5 h resulted in a significant reduction in lignin content, regardless of ultrasonic frequency. The lowest lignin content (10.66 %) was recorded in the CL-B-4 sample, indicating that higher acidity in DES, coupled with prolonged treatment, enhances lignin solubilisation.

These results align with previous studies. Oh et al. [37] reported a substantial decrease in lignin content, from 28.1 % (untreated) to 16.5 % (DES-treated), after treating pine wood powder with DES (1:2 ChCl:LA, 130 °C, 6 h). The similarity in findings suggests that acidic DES efficiently disrupts lignin-carbohydrate linkages, facilitating lignin removal.

The lignin solubilisation efficiency ranged from 28.06 % to 52.78 % in CL-A DES-treated samples and 47.74 % to 70.54 % in CL-B DES-treated samples, with the highest efficiency recorded at 45 kHz for 5 h in both cases. In CL-B DES-treated samples, increasing treatment time (3 to 5 h) at a constant ultrasonic frequency significantly enhanced lignin solubilisation. Similarly, at a constant treatment time (3 or 5 h), increasing ultrasonic frequency (25 to 45 kHz) further improved lignin removal efficiency, highlighting the synergistic role of ultrasound and acidic DES in lignin dissolution.

The lignin solubilisation efficiency observed in this study was comparable to or higher than previously reported values. Hou et al. [40] achieved 51.3 % lignin removal from rice straw using DES (1:1 ChCl:LA, 80 °C, 6 h). Chourasia et al. [36] reported lignin removal between 62.25 % and 81.63 % in sugarcane bagasse treated with DES (1:5 ChCl:LA, 80 °C, 12 h). Similarly, Yu et al. [38] found that *Triarrhena lutarioriparia* treated with DES at 90 °C for 3 h exhibited lignin solubilisation of 23.85 % (1:2 ChCl:LA) and 43.66 % (1:10 ChCl:LA). These results confirm that lignin removal efficiency is influenced by multiple factors, including pretreatment conditions, biomass type, and DES composition.

The ash content in the solid fraction recovered after US-DES treatment ranged from 3.59 % to 3.72 % for samples treated with CL-A DES, and from 5.05 % to 6.95 % for samples treated with CL-B DES. The CL-B DES-treated samples had lower hemicellulose and lignin content compared to the CL-A DES-treated samples, which may have contributed to the slightly higher ash content observed in the CL-B DES-treated samples.

The effectiveness of DES pretreatment depends on its ability to disrupt the lignin-carbohydrate complex in biomass. This efficiency is governed by molecular interactions between the hydrogen bond donor (HBD) and hydrogen bond acceptor (HBA) components of DES [41]. Acidic DES is particularly effective in breaking down the lignin-carbohydrate matrix, as biomass with high lignin content typically contains a significant proportion of β-O-4-aryl ether bonds, which are highly susceptible to acidic cleavage [36,42]. Additionally, Oh et al. [37] proposed that DES with higher hydrogen bond acidity enhances lignin solubilisation, primarily by catalysing the cleavage of β-O-4 ether bonds, thereby facilitating lignin removal.

Hemicellulose, being more reactive than cellulose, can be selectively dissolved and separated from cellulose-rich fractions during acidic DES treatment. Its higher solubility is attributed to its shorter chain length (degree of polymerisation: ∼100–200) compared to cellulose (10,000–14,000), as well as its distinct ring structures and hydroxyl configurations [43]. This differential solubility enables the preferential extraction of hemicellulose, leaving behind a cellulose-enriched solid residue.

Furthermore, the integration of ultrasonication in DES pretreatment significantly enhances biomass-solvent interactions by promoting dynamic contact between the solvent and biomass. The ultrasound waves induce cavitation, causing the formation and collapse of microscopic bubbles in the liquid medium (DES in this case). This process generates localised high-energy densities, enhancing solid–liquid mass transfer and biomass fragmentation. The rapid collapse of cavitation bubbles leads to the formation of local hotspots, where the energy release occurs faster than dissipation, resulting in high temperatures and intense micro-scale turbulence. These conditions contribute to disrupting the crystalline structure of biomass, thereby accelerating reaction rates and improving the overall efficiency of DES-based pretreatment [41].

#### 3.1.2 Cellulose purification

Following US-DES treatment, the CL-B-4 sample (45 kHz frequency, 5-hour treatment time) was selected for further downstream processing, as it provided an optimal balance between key pretreatment efficiency parameters, including maximum cellulose retention in the recovered solid fraction and high solubilisation efficiency of both hemicellulose and lignin. The purification of the DES-treated solid residue obtained from the CL-B-4 sample resulted in a purified cellulose fibre (PCF) yield (CL-B-PCF) of 39.81 ± 1.47 %. The purity of cellulose in CL-B-PCF was measured at 91.31 ± 0.93 %. In the CL-B-4 sample, non-cellulosic components (hemicellulose, lignin, and ash) constituted an average of 30.34 %. After purification, the proportion of these components in PCF decreased to an average of 8.69 %, indicating a substantial reduction in non-cellulosic materials. The cellulose yield and purity obtained from barley straw in this study were comparable to those reported for similar lignocellulosic biomass in the literature.

For instance, Freitas et al. [44] employed a green extraction method combining ultrasound and reflux heating, followed by bleaching, to extract cellulose fibres from rice straw, achieving a cellulose yield of 37.4 % with an average purity of 65.9 %. Similarly, Qu et al. [45] extracted cellulose from wheat straw using a three-step method consisting of ultrasonic pretreatment, bleaching, and alkaline treatment, reporting a cellulose yield ranging from 32.80 % to 35.73 % and purity levels between 75.26 % and 86.50 %, depending on ultrasonic treatment time.

The higher cellulose purity (91.31 %) obtained in this study compared to these previous works suggests that the US-DES and purification processes used were highly effective in removing hemicellulose and lignin, leading to a high-purity cellulose fraction suitable for nanocellulose production.

#### 3.1.3 Recovery of lignin and DES

Following US-DES pretreatment, the liquid fraction primarily contained lignin, hemicellulose, and DES. Lignin was recovered from this fraction using water as an anti-solvent, a process based on the principle that lignin solubility decreases with increasing water content in DES [37]. This selective precipitation mechanism enables the separation of lignin from hemicellulose, facilitating its recovery in solid form. The lignin-rich material (LRM) was recovered from the CL-B-4 sample following the method described in Section 2.4.3. The LRM yield was 9.40 ± 0.89 %, with a lignin purity of 83.29 ± 1.57 %. Additionally, the lignin recovery efficiency was 34.50 ± 2.68 %, indicating effective lignin fractionation under the selected pretreatment conditions.

The lignin recovery efficiency observed in this study was comparable to previously reported values. Oh et al. [37] achieved 53 % lignin recovery with over 90 % purity using DES (1:2 ChCl:LA) treatment of pine wood powder at 130 °C for 6 h. Similarly, Suopajärvi et al. [43] reported lignin yields of 11.8 wt-% from rapeseed stem, 9.5 wt-% from corn stalk, and 8.5 wt-% from wheat straw, following DES (1:5 ChCl:LA) treatment at 100 °C for 16 h. The slightly lower lignin recovery in this study may be attributed to differences in biomass composition, treatment duration, and DES molar ratios. The recycling rate of DES is a critical factor influencing the economic feasibility of DES-based biorefinery processes, as solvent recovery significantly impacts operational costs [46]. The DES recovery rate from the CL-B-4 sample was 83.97 ± 3.42 %, demonstrating the potential for solvent reuse in subsequent processing cycles.

Comparable findings have been reported in the literature. Satlewal et al. [47] achieved a DES recovery rate of ∼ 88 % after the first cycle of sugarcane bagasse pretreatment using DES (1:5 ChCl:LA). Similarly, Shen et al. [46] reported a 90 % recovery rate for DES (1:10 ChCl:LA) after Eucalyptus sawdust pretreatment, indicating that higher water content in DES formulations may enhance recyclability. The high DES recovery rate observed in this study suggests that US-DES pretreatment is not only effective but also economically viable, supporting its application in sustainable lignocellulosic biorefineries.

### 3.2 Hydrated DES treatment of PCF

The CL-B-PCF sample was treated with BOW HDES under varying cellulose treatment factors (CTF), including different autoclave temperatures, ramp-up times, holding times, and cooling times. The recovery rates of CL-B-PCF after treatment with three different HDES variants (BOW-1, BOW-2, and BOW-3) are presented in Table 4. For all three HDES types, a decrease in CL-B-PCF recovery was observed as autoclave holding time increased, irrespective of the treatment temperature. This finding suggests that longer exposure times led to greater solubilisation of PCF in HDES, likely due to extended interaction between cellulose and solvent components. Additionally, higher treatment temperatures (120 °C) resulted in lower recovery rates compared to lower temperatures (100 °C) for the same holding time across all HDES formulations. This highlights that temperature significantly influences the solubilisation rate of PCF in HDES, regardless of the specific solvent composition.

**Table 4:** BOW HDES treatment of CL-B-PCF cellulose sample at different cellulose treatment factors (CTF) with a cellulose to HDES ratio of 1:25 w/v.

Cellulose treatment factors	Temperature (°C)	Treatment time in autoclave (min)			Recovery of CL-B-PCF after HDES treatment (%)		
		Ramp-up time	Holding time	Cooling time	BOW-1	BOW-2	BOW-3
CTF-1	100	20	30	20	93.05 ± 1.63^e^	93.16 ± 1.67^e^	91.07 ± 1.58^e^
CTF-2	100	20	60	20	89.37 ± 0.94^d^	88.57 ± 1.10^d^	87.50 ± 1.01^d^
CTF-3	100	20	90	20	86.98 ± 1.06^d^	87.32 ± 1.30^cd^	84.58 ± 1.07^d^
CTF-4	100	20	120	20	82.78 ± 1.23^c^	83.75 ± 1.15^c^	78.79 ± 1.56^c^
CTF-5	120	25	30	25	90.55 ± 1.89^de^	89.23 ± 1.74^d^	86.84 ± 1.06^d^
CTF-6	120	25	60	25	79.97 ± 1.20^bc^	79.55 ± 2.15^b^	78.34 ± 0.94^bc^
CTF-7	120	25	90	25	78.30 ± 0.99^b^	77.78 ± 0.95^b^	75.18 ± 1.72^ab^
CTF-8	120	25	120	25	74.27 ± 1.17^a^	73.37 ± 1.15^a^	73.58 ± 0.71^a^

*Different letters in the same column indicate statistically significant differences (P < 0.05); n = 3.

Among the three HDES variants, BOW-1 and BOW-2 exhibited similar treatment efficiency, with comparable CL-B-PCF recovery rates. In contrast, BOW-3 HDES resulted in a slightly lower recovery rate than BOW-1 and BOW-2, though the difference was statistically insignificant. This suggests that variations in HDES composition marginally affect cellulose solubilisation under the tested conditions. The optimal CTF was determined based on the objective of achieving a balanced hydrolysis efficiency, ensuring that the extent of hydrolysis was neither too low nor excessive. The CL-B-PCF recovery rate across all HDES treatments ranged from 73 % to 93 %. To maintain controlled hydrolysis, a target recovery rate of ∼ 80 % was set, preventing excessive degradation while allowing sufficient structural modification. The CTF-6 sample (120 °C, 25-minute ramp-up time, 60-minute holding time, and 25-minute cooling time) closely matched this target recovery rate. The average CL-B-PCF recovery for BOW-1, BOW-2, and BOW-3 HDES was 79.97 %, 79.55 %, and 78.34 %, respectively. Based on these findings, CTF-6 was selected for further downstream processing with HIUS treatment for nanocellulose production.

These findings align with previous research on HDES-mediated cellulose modification. In a comparable study, Hardiningtyas et al. [48] investigated the effect of hydrated DES (HDES) on cellulose extracted from industrial agar seaweed waste biomass. They added distilled water to 100 % ChCl-based DES (ChCl:urea, ChCl:citric acid, and ChCl:oxalic acid) to achieve a 10 % v/v DES concentration. Following HDES treatment, the cellulose underwent ultrasonication and homogenisation to produce nanocellulose. Their study demonstrated that cellulose hydrolysis in HDES led to the formation of nanocellulose or even reducing sugars, highlighting the importance of selecting an appropriate solvent system. Furthermore, they observed that nanocellulose yield was influenced by the solubility properties of the HDES, reinforcing the significance of solvent composition in the cellulose modification process [48].

### 3.3 Characterization of nanocellulose

#### 3.3.1 DLS analysis

**Fig. 1:**
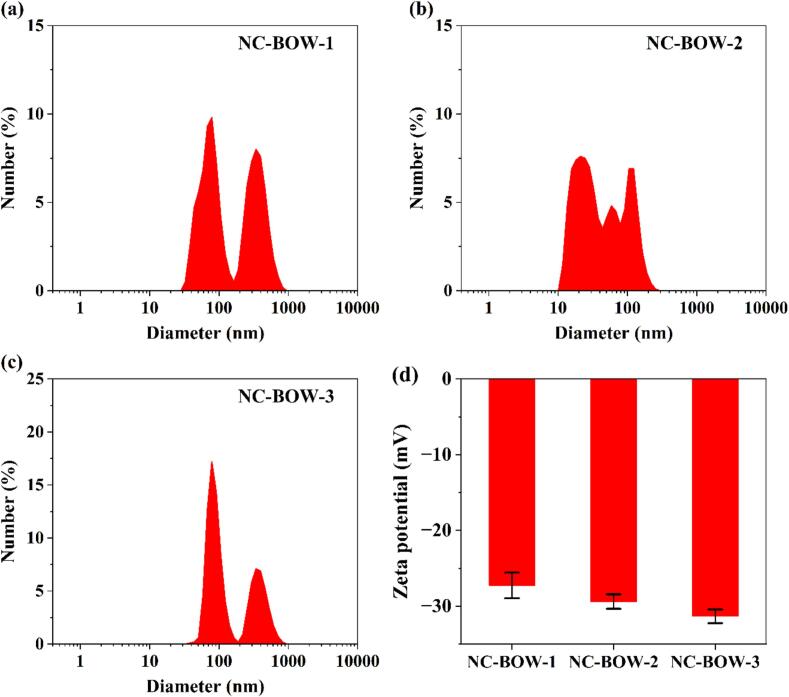
Number size distribution plots and zeta potential of NC-BOW nanocellulose samples.

Dynamic Light Scattering (DLS) is a widely used, non-destructive technique for measuring particle size [49]. However, a limitation of DLS technique is its assumption that all particles are spherical [50,51]. Nonetheless, the technique has been utilised for measurement of particle size of nanocellulose sample in several recent studies [[52], [53], [54]]. The DLS analysis was conducted to determine the particle size distribution, polydispersity index (PDI), and zeta potential of the nanocellulose suspension. The number size distribution of the three nanocellulose samples (NC-BOW-1, NC-BOW-2, and NC-BOW-3) is depicted in Fig. 1a, 1b, and 1c, respectively. The distribution plots were generated using average values from nine measurements across three distinct samples.

For the NC-BOW-1 nanocellulose sample, approximately 46.4 % of nanoparticles had a diameter of less than 100 nm, while 9 % of nanoparticles fell within the 100–200 nm range, and 43.5 % of nanoparticles were in the 200–1000 nm range. Similarly, for NC-BOW-2, around 77.8 % of nanoparticles were below 100 nm, with 21.4 % between 100 and 200 nm, and only 0.5 % between 200 and 1000 nm. For NC-BOW-3, 49.7 % of nanoparticles were below 100 nm, while 15.1 % were between 100 and 200 nm, and 32.6 % were within 200–1000 nm.

Numerous studies have utilised DLS analysis to examine the particle size distribution of nanocellulose derived from biomass sources similar to barley straw. For instance, Jirathampinyo et al. [55] reported that nanocellulose produced from rice straw cellulose via enzymatic hydrolysis and ultrasonication-assisted pretreatment had a particle size distribution ranging from 140 to 955 nm. Trivedi et al. [56] extracted nanocellulose from wheat straw through chemical pretreatments, followed by grinding and sieving, yielding nanocellulose particles between 13 and 50 nm, with 90 % of the nanoparticles measuring ∼ 30 nm. Similarly, Bhardwaj et al. [57] extracted cellulose nanocrystals (CNCs) from mustard straw using acid hydrolysis followed by sonication, obtaining a mean particle size of 584.71 nm. The findings from the current study, along with literature reports, suggest that particle size distribution is influenced by multiple factors, including biomass type, pretreatment conditions, and production methods.

The PDI value provides insight into particle size uniformity, with monodisperse samples exhibiting a PDI of zero, while values approaching 1 indicate a broader size distribution [58]. The PDI values for NC-BOW-1, NC-BOW-2, and NC-BOW-3 were 0.526 ± 0.064, 0.395 ± 0.095, and 0.519 ± 0.109, respectively. The NC-BOW-2 nanocellulose sample exhibited a significantly lower PDI than NC-BOW-1 and NC-BOW-3, suggesting that NC-BOW-2 had a more uniform size distribution. In contrast, NC-BOW-1 and NC-BOW-3 displayed a broader particle size distribution, which was consistent with their respective number size distribution profiles. Similar findings have been reported by Aguiar et al. [58], who observed PDI values between 0.303 and 0.364 for CNC extracted from sugarcane straw via enzymatic deconstruction, while CNC derived from sugarcane bagasse exhibited a PDI range of 0.272 to 0.329.

The zeta potential is a key indicator of colloidal stability, with values above + 30 mV or below − 30 mV suggesting a stable suspension due to electrostatic repulsion [55]. The zeta potential values for all three nanocellulose samples are presented in Fig. 1d. The measured values were close to − 30 mV, confirming the colloidal stability of the nanocellulose suspensions. Among the three samples, NC-BOW-3 exhibited the lowest zeta potential; however, no significant difference was observed between NC-BOW-2 and NC-BOW-3. The NC-BOW-3 nanocellulose was prepared from the CL-B-PCF sample treated with BOW-3 HDES, which contained a higher proportion of betaine and oxalic acid compared to BOW-1 and BOW-2 HDES. The treatment with BOW-3 HDES likely introduced a greater number of functional groups, affecting the surface charge and electrostatic interactions of the nanoparticles, and resulting in a lower zeta potential. Similar zeta potential values have been reported for nanocellulose extracted from comparable biomass sources, including rice straw (−29.7 mV) [59], quinoa straw (−31.34 mV) [60], sugarcane straw (−25 mV) [58], and soybean straw (−24.5 mV for cellulose nanofibrils (CNF) and − 28.8 mV for CNC) [61]. Considering that NC-BOW-2 exhibited the highest proportion of particles below 100 nm, the lowest PDI, and a stable zeta potential, it was selected as the optimal nanocellulose sample. This sample was subsequently characterised using Fourier transform infrared (FTIR) spectroscopy and X-ray diffraction (XRD) techniques to assess its chemical composition and crystallinity.

#### 3.3.2 FTIR

**Fig. 2:**
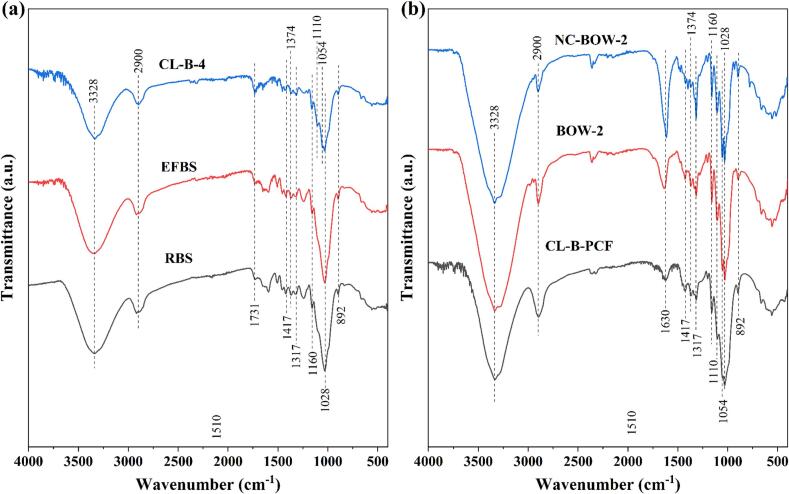
FTIR spectra of (a) RBS, EFBS, and CL-B-4 samples; (b) CL-B-PCF, BOW-2 and NC-BOW-2 samples.

FTIR spectroscopy was used to identify functional groups and structural changes occurring during the nanocellulose synthesis process from biomass [62]. The FTIR spectra of RBS, EFBS, and CL-B-4 samples are presented in Fig. 2a, while those of CL-B-PCF, BOW-2 treated, and NC-BOW-2 samples are shown in Fig. 2b. All samples exhibited characteristic peaks at approximately 3328 cm^−1^ and 2900 cm^−1^, which correspond to the stretching vibrations of hydroxyl (OH) groups and C–H bonds, respectively [63]. A distinct peak at 1731 cm^−1^ was observed in RBS, EFBS, and CL-B-4, indicating the presence of carbonyl (C=O) groups, which are commonly found in acetyl groups and uronic acids in hemicelluloses. This peak may also be associated with ester linkages in carboxylic groups of p-coumaric acid or ferulic acid within lignin [64]. The disappearance of this peak in CL-B-PCF, BOW-2, and NC-BOW-2 confirmed that most lignin and hemicellulose were successfully removed following US-DES and HDES treatments, validating the effectiveness of these processes.

A peak at 1630 cm^−1^, corresponding to the bending vibration of absorbed water, was detected in CL-B-PCF, BOW-2, and NC-BOW-2 samples [25]. The intensity of this peak was higher in BOW-2 and NC-BOW-2 compared to CL-B-PCF, suggesting that HDES treatment enhanced molecular interactions between cellulose and water, thereby increasing the hydrophilicity of the nanocellulose. Additionally, several weaker absorption bands were observed at 1417 cm^−1^, 1374 cm^−1^, and 1317 cm^−1^, corresponding to C–H stretching and C–H or O–H bending vibrations. A peak at 892 cm^−1^ was associated with the C-1 group or ring frequency, a characteristic feature of β-glycosidic linkages in cellulose [65]. Further, the peaks at 1028 cm^−1^ and 1054 cm^−1^ were attributed to C–O–C stretching of β-1,4-glycosidic bonds and C–O stretching, respectively, both of which are defining structural features of cellulose [51]. Peaks at 1110 cm^−1^ and 1160 cm^−1^ were assigned to C–O and C–O–C stretching vibrations, associated with the pyranose ring structure in polysaccharides [25,66,67].

Notably, the peaks at 1054 cm^−1^ and 1110 cm^−1^ were present in CL-B-4, CL-B-PCF, BOW-2, and NC-BOW-2, but absent in RBS and EFBS, indicating that these cellulose-specific signals became more pronounced following US-DES pretreatment and subsequent purification steps. These results confirm that the applied biorefinery process efficiently removed non-cellulosic components, enhancing cellulose purity and supporting nanocellulose production with well-defined structural integrity.

#### 3.3.3 XRD

**Fig. 3:**
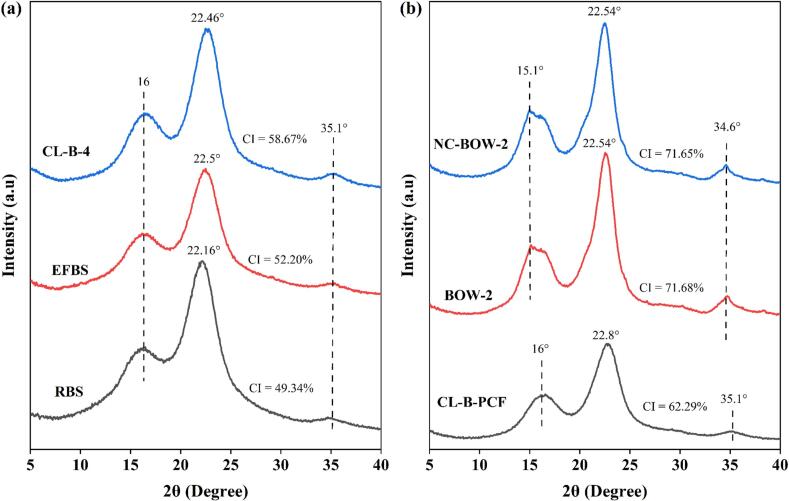
XRD analysis of (a) RBS, EFBS, and CL-B-4 samples; (b) CL-B-PCF, BOW-2 and NC-BOW-2 samples.

The XRD patterns of RBS, EFBS, and CL-B-4 samples are presented in Fig. 3a, while those of CL-B-PCF, BOW-2 treated, and NC-BOW-2 samples are shown in Fig. 3b. In all samples, a strong and sharp diffraction peak was observed between the 2θ angles of 22.16° and 22.8°, corresponding to the (200) crystalline plane, which is a characteristic feature of cellulose I [35,68,69]. Additionally, for the RBS, EFBS, CL-B-4, and CL-B-PCF samples, a peak at 2θ = 16° was detected, representing the (110) crystallographic plane, which further confirms the presence of cellulose I structure [70,71]. However, for the BOW-2 and NC-BOW-2 samples, a peak appeared at 15.1°, which corresponds to the (1 − 10) crystalline plane and is similarly associated with cellulose I [72]. Moreover, a small diffraction peak was detected at ∼ 35.1° in RBS, EFBS, CL-B-4, and CL-B-PCF samples, while a similar peak at 34.6° was present in BOW-2 and NC-BOW-2 samples. This diffraction feature near 35° is attributed to the (004) crystalline plane, further confirming the dominance of cellulose I structure in all samples [[70], [71], [72], [73]].

The crystallinity index (CI) of all samples was determined using Segal’s method, and the values were found to be 49.34 % for RBS, 52.20 % for EFBS, 58.67 % for CL-B-4, 62.29 % for CL-B-PCF, 71.68 % for BOW-2, and 71.65 % for NC-BOW-2. A progressive increase in CI was observed after each processing step, which can be attributed to the removal of amorphous components, including lignin, hemicellulose, and other non-cellulosic materials [62,71]. The increase in CI is a well-documented phenomenon in cellulose isolation processes, as the elimination of less-ordered amorphous regions enhances the relative proportion of the crystalline cellulose fraction. Notably, no significant difference in CI was observed between BOW-2 and NC-BOW-2 samples, indicating that the final HIUS treatment did not significantly alter crystallinity. This suggests that HIUS primarily contributes to particle size reduction without affecting the fundamental crystalline structure of cellulose.

The CI value of nanocellulose (NC-BOW-2) obtained from RBS in this study was comparable to values reported for nanocellulose produced from similar biomass sources, such as soybean straw (67 %) [74], wheat straw (76.43 %) [75], rice straw (76.99 %) [76], rapeseed straw (58 % to 79.41 %) [77], and sugarcane straw (62.66 %) [78]. The high crystallinity of nanocellulose is particularly advantageous for applications requiring enhanced mechanical strength, thermal stability, and resistance to enzymatic degradation. The results confirm that the US-DES and HDES treatments were highly effective in progressively increasing cellulose crystallinity, making the obtained nanocellulose suitable for advanced material applications where a highly crystalline structure is desirable.

#### 3.3.4 Scanning electron microscopy (SEM)

**Fig. 4:**
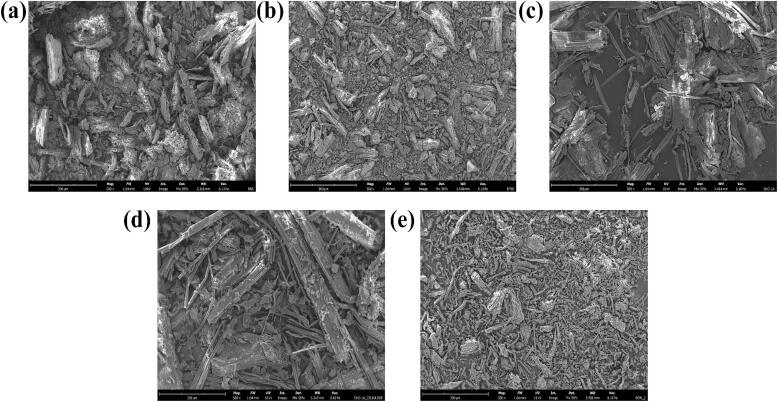
SEM images of (a) RBS; (b) EFBS; (c) CL-B-4; (d) CL-B-PCF and (e) BOW-2 samples at magnification of 500 x.

The SEM images of RBS, EFBS, CL-B-4, CL-B-PCF, and BOW-2 treated samples are presented in Fig. 4, illustrating the morphological changes occurring at different stages of nanocellulose production. The SEM image of RBS (Fig. 4a) exhibited a rough and irregular structure, where biomass components such as lignin, cellulose, hemicellulose, and non-lignocellulosic materials appeared to be aggregated. This compact and non-uniform surface suggests the presence of a complex lignocellulosic network, characteristic of untreated plant biomass.

Following the removal of non-lignocellulosic components, the SEM image of EFBS (Fig. 4b) revealed a smoother morphology with the presence of pores and voids compared to RBS. These structural modifications can be attributed to the extraction of ethanol- and water-soluble impurities, which may have contributed to partial loosening of the biomass matrix. The formation of pores and voids indicates enhanced accessibility of the cellulose fibrils, facilitating subsequent DES-based treatments.

In the CL-B-4 sample (Fig. 4c), a more filamentous and smooth structure was observed, with the presence of individual cylindrical and rod-shaped cellulose fibrils, as well as some cellulosic bundles. However, the cellulose fibrils appeared to be partially coated with residual impurities, likely traces of lignin and/or hemicellulose. This observation is in agreement with the composition analysis results of the CL-B-4 sample, where residual lignin and hemicellulose were still present in the solid fraction (Table 3).

Following purification, the SEM image of CL-B-PCF (Fig. 4d) showed that both individual and bundled cellulose fibrils appeared to be more refined and freer from visible impurities. However, the fibrils displayed non-homogeneous sizes and shapes, with some larger fibril aggregates present. This suggests that while lignin and hemicellulose were effectively removed, mechanical fibrillation during processing was not completely uniform, potentially leading to partial aggregation of cellulose fibrils.

After the BOW HDES treatment under CTF-6 conditions, the SEM image of BOW-2 (Fig. 4d) exhibited a well-separated and fine fibrillar structure, with cellulose fibres appearing more homogenous in size and shape. This enhanced fibrillation may be attributed to HDES-induced hydrolysis at elevated temperatures, which could have partially cleaved hydrogen bonds within the cellulose microfibrils, promoting better individualisation of cellulose fibres. These findings confirm that BOW HDES treatment effectively improved cellulose fibrillation, making it a promising method for obtaining uniformly fibrillated cellulose suitable for nanocellulose production.

### 3.4 Life cycle assessment

The environmental impact of each unit process (UP) involved in the production of nanocellulose from barley straw was assessed. In the system boundary (Fig. S1), each unit process was coded as follows: (i) UP-A: Grinding of Barley straw (RBS), (ii) UP-B: Extraction of non-lignocellulosic components (EFBS), (iii) UP-C: Pretreatment of biomass (CL-B-4), (iv) UP-D: Lignin rich material (LRM) and DES recovery, (v) UP-E: Cellulose purification (CL-B-PCF), (vi) UP-F: HDES treatment of cellulose (BOW-2 HDES at CTF-6), and (vii) UP-G: HIUS treatment of HDES treated cellulose for nanocellulose production (NC-BOW-2).

The environmental impact of each UP along with the overall impact of the process is provided in Table 5. For climate change category, the total impact was 6.547 kg CO_2_ eq, predominantly driven by unit processes UP-B (2.601 kg CO_2_ eq) and UP-C (2.512 kg CO_2_ eq), contributing 40 % and 38 %, respectively. Freshwater ecotoxicity category showed a total impact of 22.425 CTUe, with UP-C (9.773 CTUe) and UP-B (7.373 CTUe) being the largest contributors, accounting for 44 % and 33 %, respectively. The land use category had a total impact of 21.133Pt, with UP-B (8.682Pt) and UP-C (7.863Pt) contributing significantly, accounting for 41 % and 37 %, respectively. The resource use (fossils) was another major damage category which had a total impact of 105.225 MJ, with UP-B (41.833 MJ) and UP-C (40.905 MJ) being the highest contributors, accounting for 40 % and 39 %, respectively. Another key damage category identified was water use which had a total impact of 1.197 m^3^ depriv., with UP-C (0.484 m^3^ depriv.) and UP-B (0.397 m^3^ depriv.) being the main contributors, accounting for 40 % and 33 %, respectively.

**Table 5:** Environmental impact assessment of nanocellulose production process at a laboratory scale.

**Damage category / Cost**	**Unit**	**Total**	**UP-A**	**UP-B**	**UP-C**	**UP-D**	**UP-E**	**UP-F**	**UP-G**
Acidification	mol H + eq	0.025	0.000	0.009	0.009	0.001	0.001	0.004	0.000
Climate change	kg CO_2_ eq	6.547	0.007	2.601	2.512	0.382	0.282	0.707	0.055
Ecotoxicity, freshwater	CTUe	22.425	0.020	7.373	9.773	1.092	0.858	3.074	0.235
Particulate matter	disease inc.	0.000	0.000	0.000	0.000	0.000	0.000	0.000	0.000
Eutrophication, marine	kg N eq	0.005	0.000	0.001	0.002	0.000	0.000	0.001	0.000
Eutrophication, freshwater	kg P eq	0.000	0.000	0.000	0.000	0.000	0.000	0.000	0.000
Eutrophication, terrestrial	mol N eq	0.051	0.000	0.016	0.017	0.002	0.002	0.014	0.000
Human toxicity, cancer	CTUh	0.000	0.000	0.000	0.000	0.000	0.000	0.000	0.000
Human toxicity, non-cancer	CTUh	0.000	0.000	0.000	0.000	0.000	0.000	0.000	0.000
Ionising radiation	kBq U-235 eq	0.158	0.000	0.064	0.059	0.010	0.007	0.016	0.001
Land use	Pt	21.133	0.023	8.682	7.863	1.277	0.950	2.153	0.186
Ozone depletion	kg CFC11 eq	0.000	0.000	0.000	0.000	0.000	0.000	0.000	0.000
Photochemical ozone formation	kg NMVOC eq	0.017	0.000	0.006	0.007	0.001	0.001	0.003	0.000
Resource use, fossils	MJ	105.225	0.110	41.833	40.905	5.967	4.403	11.145	0.863
Resource use, minerals and metals	kg Sb eq	0.000	0.000	0.000	0.000	0.000	0.000	0.000	0.000
Water use	m^3^ depriv.	1.197	0.001	0.397	0.484	0.074	0.042	0.121	0.078

UP-A: Grinding of Barley straw (RBS); UP-B: Extraction of non-lignocellulosic components (EFBS); UP-C: Pretreatment of biomass (CL-B-4); UP-D: Lignin rich material (LRM) and DES recovery; UP-E: Cellulose purification (CL-B-PCF); UP-F: HDES treatment of cellulose (BOW-2 HDES at CTF-6); UP-G: HIUS treatment of HDES treated cellulose for nanocellulose production (NC-BOW-2).

The damage categories such as acidification, eutrophication (terrestrial), ionizing radiation, photochemical ozone formation showed minor environmental impacts. On the other hand, the damage categories such as particulate matter, eutrophication (freshwater and marine), human toxicity (cancer and non-cancer), ozone depletion, and resource use (minerals and metals) showed negligible impacts, indicating that they were not significant contributors to the overall environmental impact of the process.

Overall, the data indicated that the unit processes UP-B and UP-C were the most significant contributors across multiple environmental impact categories. These two unit processes could be the primary limitations when it comes to scaling up the developed nanocellulose production process to an industrial level. In addition, replicating the quality of products achieved at the laboratory scale poses another limitation for scaling up the developed process to an industrial level, as the equipment used in an industrial facility may differ.

## 4 Conclusion

This study successfully developed an efficient and sustainable biorefinery approach for the valorisation of agricultural waste biomass, integrating ultrasound-assisted deep eutectic solvent (DES) pretreatment, cellulose purification, and nanocellulose production using a sequential hydrated DES (HDES) treatment followed by high-intensity ultrasound (HIUS) treatment. The process was further optimised by incorporating lignin recovery and DES recycling, enhancing both resource efficiency and economic feasibility. The pretreatment process achieved close to 70 % solubilisation of lignin and hemicellulose, leading to an enriched cellulose fraction with a yield of 39.81 % after purification. Additionally, 9.40 % of lignin-rich material was successfully recovered, demonstrating the potential of this approach for lignin valorisation.

The optimised nanocellulose production process resulted in 77.8 % of nanoparticles with diameters below 100 nm, confirming effective fibrillation. Moreover, the nanocellulose exhibited good colloidal stability in aqueous suspension, with a zeta potential close to − 30 mV, ensuring long-term dispersion stability.

This study demonstrates that the developed biorefinery strategy effectively converts agricultural residues into high-value bioproducts, with DES-based pretreatment, solvent recovery, and optimised nanocellulose production supporting sustainable bioeconomy solutions. The nanocellulose could be further investigated in various potential applications such as development of food packaging materials, nanocomposite materials, membranes for water purification and many others.

In future work, the efficiency each unit process could be investigated in a pilot scale set up. This would involve evaluating the consistency and quality of the purified cellulose fibres, lignin, and nanocellulose. Furthermore, monitoring resource consumption and conducting life cycle assessments at a pilot scale would provide valuable insights into the sustainability aspects of the production process. These evaluations would help make informed decisions regarding the scalability of the process.

## CRediT authorship contribution statement

**Dileswar Pradhan:** Writing – review & editing, Writing – original draft, Methodology, Investigation, Data curation, Conceptualization. **Swarna Jaiswal:** Writing – review & editing, Validation, Supervision, Conceptualization. **Brijesh K. Tiwari:** Writing – review & editing, Resources. **Amit K. Jaiswal:** Writing – review & editing, Validation, Supervision, Funding acquisition, Conceptualization.

## Declaration of competing interest

The authors declare that they have no known competing financial interests or personal relationships that could have appeared to influence the work reported in this paper.
