# Supplementary material for: Ultrasound-Assisted sequential processing of barley straw using binary acidic and hydrated ternary deep eutectic solvents for nanocellulose production
Source: Ultrason Sonochem. 2025 May 5;118:107376. doi: 10.1016/j.ultsonch.2025.107376 (PMC12147838; doi:10.1016/j.ultsonch.2025.107376)
Supplement: Supplementary Data 1 [file mmc1.pdf]

Supplementary Materials

Ultrasound-Assisted Sequential Processing of Barley Straw Using Binary Acidic and Hydrated Ternary Deep Eutectic Solvents for Nanocellulose Production

Dileswar Pradhan[a,b,c,d], **Swarna Jaiswal**[a,b,c,d], **Brijesh K. Tiwari**[e], **Amit K. Jaiswal**[a,b,c,d,*]

[a]*School of Food Science and Environmental Health, Faculty of Sciences and Health, Technological University Dublin - City Campus, Central Quad, Grangegorman, Dublin, Ireland*

[b]*Centre for Sustainable Packaging and Bioproducts, Technological University Dublin - City Campus, Central Quad, Grangegorman, Dublin, Ireland*

[c]*Sustainability and Health Research Hub, Technological University Dublin - City Campus, Grangegorman, Dublin, Ireland*

[d]*Health Engineering & Materials Science Research Hub, Technological University Dublin - City Campus, Grangegorman, Dublin, Ireland*

[e]*Teagasc Food Research Centre, Ashtown, Dublin, Ireland*

*Corresponding author: amit.jaiswal@tudublin.ie (https://orcid.org/0000-0002-4551-4182)

E-mails of other authors:

Dileswar Pradhan : dileswar.pradhan@tudublin.ie (https://orcid.org/0000-0001-6102-2236)

Swarna Jaiswal : swarna.jaiswal@tudublin.ie (https://orcid.org/0000-0003-1414-9052)

Brijesh K. Tiwari : brijesh.tiwari@teagasc.ie (https://orcid.org/0000-0002-4834-6831)

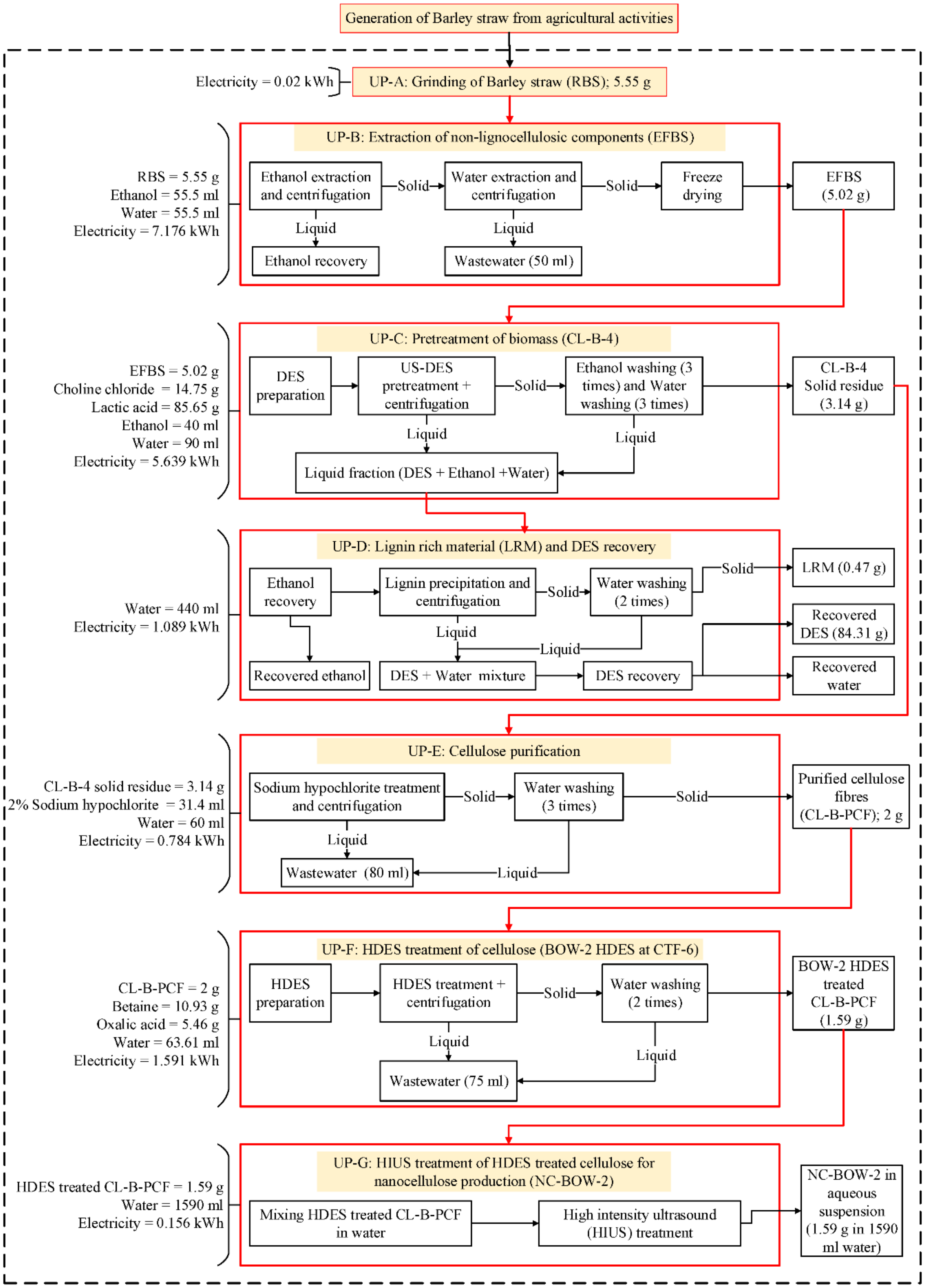

Figure S1. System boundary of the nanocellulose production process for life cycle assessment.
